# An efficient system for the generation of marked genetic mutants in members of the genus *Burkholderia*

**DOI:** 10.1016/j.plasmid.2016.11.002

**Published:** 2017-01

**Authors:** Sravanthi Shastri, Helena L. Spiewak, Aderonke Sofoluwe, Vigdis A. Eidsvaag, Atif H. Asghar, Tyrone Pereira, Edward H. Bull, Aaron T. Butt, Mark S. Thomas

**Affiliations:** Department of Infection, Immunity and Cardiovascular Disease, The Medical School, The University of Sheffield, Beech Hill Road, Sheffield S10 2RX, UK,

**Keywords:** BHR, broad host-range, MCS, multiple cloning site, TIR, translation initiation region, Cm^R^, chloramphenicol resistance/resistant, Cm^S^, chloramphenicol sensitive, Km^R^, kanamycin resistance, Tc^R^, tetracycline resistance, Tp^R^, trimethoprim resistance, Marked mutation, Gene inactivation, *Burkholderia cepacia* complex, Suicide vector, Antibiotic-resistance

## Abstract

To elucidate the function of a gene in bacteria it is vital that targeted gene inactivation (allelic replacement) can be achieved. Allelic replacement is often carried out by disruption of the gene of interest by insertion of an antibiotic-resistance marker followed by subsequent transfer of the mutant allele to the genome of the host organism in place of the wild-type gene. However, due to their intrinsic resistance to many antibiotics only selected antibiotic-resistance markers can be used in members of the genus *Burkholderia*, including the *Burkholderia cepacia* complex (Bcc). Here we describe the construction of improved antibiotic-resistance cassettes that specify resistance to kanamycin, chloramphenicol or trimethoprim effectively in the Bcc and related species. These were then used in combination with and/or to construct a series enhanced suicide vectors, pSHAFT2, pSHAFT3 and pSHAFT-GFP to facilitate effective allelic replacement in the Bcc. Validation of these improved suicide vectors was demonstrated by the genetic inactivation of selected genes in the Bcc species *Burkholderia cenocepacia* and *B. lata*, and in the non-Bcc species, *B. thailandensis*.

## Highlights

• We have constructed antibiotic-resistance cassettes and suicide vectors for use in *Burkholderia* and related species.
• These vectors facilitate construction of mutants by gene disruption with antibiotic-resistance markers.
• We have validated the utility of the vectors for marked genetic inactivation in members of the genus *Burkholderia*.

## 1 Introduction

*Burkholderia cepacia* complex (Bcc) are a group of at least twenty closely related Gram-negative bacterial species of the *Burkholderia* genus, which are phenotypically similar but genetically different (Vandamme et al., 1997, De Smet et al., 2015). These species are found ubiquitously in the environment, including soil, water and the rhizosphere of some plants (Coenye and Vandamme, 2003). Many Bcc species have also been isolated from the sputum of cystic-fibrosis (CF) patients, identifying them as opportunistic pathogens of those with lung disease and weakened immune systems (Henry et al., 1997, Speert et al., 2002). Colonisation of the lungs by Bcc can result in several clinical outcomes, including chronic-infection with gradual lung deterioration and a rare adverse reaction known as cepacia syndrome, which leads to rapid lung decline, septicaemia, necrotizing pneumonia and is often fatal (Isles et al., 1984). The most prevalent causative agents of Bcc infections in CF-patients in the UK, Canada and USA are *B. cenocepacia* and *B. multivorans* (Drevinek and Mahenthiralingam, 2010, De Soyza et al., 2010, Zlosnik et al., 2015). Unfortunately, Bcc infections can be difficult to treat due to the high intrinsic resistance of these bacteria to many antibiotics. Over recent years restrictions have been placed on individuals colonized with Bcc to prevent transmission of Bcc infections, by limiting their contact with at risk members of the population (Ledson et al., 1998).

Efforts have been made to understand the virulence mechanisms used by Bcc bacteria that allow them to be successful pathogens. These include mechanisms of bacterial cell invasion, intracellular survival, quorum sensing, iron acquisition, and protein secretion (Martin and Mohr, 2000, Sokol et al., 2003, Visser et al., 2004, Mullen et al., 2007, Aubert et al., 2008, Uehlinger et al., 2009, Somvanshi et al., 2010). A key factor to aid in the characterization of these traits is the ability to manipulate the genome of Bcc species. Methods for targeted gene inactivation in *Burkholderia* have largely relied on the disruption of the target chromosomal gene with an antibiotic-resistance marker, either through employing an integrative vector (Flannagan et al., 2007, Chapalain et al., 2013) or by allelic replacement whereby the wild type gene is replaced by a copy of the target gene that is inactivated with an antibiotic-resistance cassette (Huber et al., 2001, Malott et al., 2005, Agnoli et al., 2006). In the latter, two recombination crossover events are selected for (one occurring either side of the lesion in the target gene) and no vector sequences remain on the chromosome in the mutant. Vectors for generation of such marked mutants include plasmids in the pEX18 and pEX19 series (Hoang et al., 1998), and a vector constructed in our lab, pSHAFT (Agnoli et al., 2006).

Due to their high level of intrinsic resistance to many antibiotics, the number of commonly employed resistance markers that can be used for genetic manipulation of Bcc species is generally restricted to those specifying increased resistance to trimethoprim, chloramphenicol and tetracycline. All three markers can be selected for in single copy in *B. cenocepacia* (Farmer and Thomas, 2004, Asghar et al., 2011, Ryan et al., 2013). For aminoglycoside-sensitive strains, whether naturally occurring (such as *B. cenocepacia* strain H111) or engineered, the *aac* and *aph* markers, which specify resistance to the aminoglycosides gentamycin and kanamycin, respectively, are selectable in single copy and can therefore also be used in allelic replacement experiments (Huber et al., 2001, Hamad et al., 2010, Inhülsen et al., 2012, Carlier et al., 2014).

Allelic replacement in bacteria involving disruption of a target gene with a selectable resistance marker can be problematic if the host bacterium already exhibits a high degree of intrinsic resistance to the antibiotic, thereby precluding selection of the marker. This can be ameliorated if the antibiotic-resistance gene is efficiently expressed. However, the use of strong promoters to drive high-level transcription of the marker gene can exert detrimental polar effects that include destabilisation of plasmids housing such markers or unwanted or toxic expression of downstream genes following insertion of the marker in the host genome (Stueber and Bujard, 1982, Stassi and Lacks, 1982, Schrecke et al., 2013; our published observations). In these situations the negative effects of very active promoters can be circumvented by placement of a strong transcription terminator downstream of the selectable marker gene (Gentz et al., 1981, Fellay et al., 1987). Here we describe the construction of novel antibiotic-resistance cassettes where we have employed these principles either to increase expression of the antibiotic-resistance marker to facilitate its selection in a single copy or to prevent detrimental polar effects by blocking transcriptional read through into downstream genes. These cassettes were used to build improved suicide vectors derived from the vector pSHAFT and were also used in conjunction with these vectors for allelic replacement in the Bcc and other members of the genus *Burkholderia*.

## 2 Materials and methods

### 2.1 Strains, plasmids, and growth conditions

The bacterial strains and plasmids used in this study are given in Supplementary Table S1. For cultivation of bacteria, strains were routinely grown in/on LB medium at 37 °C. For selection of trimethoprim resistance in *E. coli* isosensitest agar (Oxoid) was employed, whereas for *B. cenocepacia* M9-minimal salts agar containing 0.5% glucose was used (except where indicated). M9-minimal salts used here contained 42 mM Na_2_HPO_4_, 22 mM KH_2_PO_4_, 19 mM NH_4_Cl, 9 mM NaCl, 1 mM MgSO_4_ and 100 μM CaCl_2_. For selection of kanamycin resistance in *B. cenocepacia* Lennox agar was utilised. Antibiotics were used, when appropriate, at the following concentrations for selection of plasmids and antibiotic-resistance cassettes in *E. coli* and *Burkholderia* species, as indicated: ampicillin, 100 μg/ml (*E. coli*); kanamycin, 50 μg/ml (*E. coli* and *B. cenocepacia*); chloramphenicol, 25 μg/ml (*E. coli*) and 50 μg/ml (*B. cenocepacia*); trimethoprim, 25 μg/ml (*E. coli*, *B. cenocepacia* and *B. lata*) and 50 μg/ml (*B. thailandensis*). For *B. cenocepacia* strains exhibiting a high intrinsic resistance to trimethoprim, such as K56-2, this antibiotic was included in the medium at 100 μg/ml.

### 2.2 DNA preparation and manipulation

Recombinant DNA techniques were performed essentially as described in Sambrook et al. (1989). DNA amplification by PCR was performed as standard according to the manufacturer's instructions using KOD DNA polymerase enzyme (Millipore) and a G-storm thermocycler. Primers used in this study are indicated in Supplementary table S2, and were purchased from Eurogentec, Belgium. To extract DNA for PCR, bacterial colonies were resuspended in 200 μl TE buffer (10 mM Tris, 1.0 mM EDTA (pH 8.0)), boiled for 7 min, and then centrifuged to pellet cell debris. The supernatant was retained for use in PCR (referred to as ‘boiled lysate’). PCR products were purified from solution or by agarose gel-extraction using a QIAquick PCR purification kit (Qiagen). DNA restriction enzymes were purchased from Promega or New England Biolabs. DNA was ligated using T4 DNA ligase (Promega). 5′ DNA overhangs were filled-in using DNA I polymerase Klenow fragment (Promega). Sequencing was performed by the Core Genomic Facility at The University of Sheffield, UK.

### 2.3 Plasmid constructions

Plasmids were extracted from saturated bacterial cultures using a spin-column-based plasmid mini-prep extraction kit (Thermo Scientific). Plasmids were transferred into *E. coli* by heat‐shock assisted transformation according to Hanahan (1983).

To construct p34E-Km, the Tn*5*-derived *aphA2* gene, including its promoter (Rothstein and Reznikoff, 1981), was excised from pKNOCK-Km as a *Mlu*I fragment, end-filled with DNA polymerase I Klenow fragment and ligated between the filled *Eco*RI sites of p34E-Tp.

To generate p34E-Cm2, the *catA2* gene of the plasmid pSHAFT was amplified with primers p34E-Cmfor2 and p34E-Cmrev, which incorporated a synthetic promoter (− 10 and − 35 elements) upstream of the *catA2* TIR in the amplified product. The 823 bp amplicon was restricted with *Eco*RI and used to replace the *Eco*RI-excised Km^R^ cassette of p34E-Km.

To construct p34E-TpTer, first a 306 bp DNA fragment containing the *rrnB* T1 T2 terminators was amplified from the plasmid pEA302T with rrnBterfor and rrnBterrev and restricted with *Bam*HI and *Hin*dIII, following which it was ligated between the corresponding restriction sites of pUC18 to give pUC18Ter. Second, the megaprimer variation of SOE PCR (Perrin and Gilliland, 1990) was used to fuse the *rrnB* T1 T2 terminators to the 3′ end of *dfrB2*. To do this, the *dfrB2* gene (and native promoter) was amplified from p34E-Tp with primer Tp(forward), and the fusion primer Tp(reverse) that was complementary to the 3′ end of the *dfrB2* gene, including the stop codon, and contained a 5′ tail with 19 bp of homology to the upstream region of the *rrnB* T1 T2 DNA fragment. The resultant amplicon served as a forward megaprimer in combination with reverse primer rrn(R), to amplify a 903 bp *dfrB2*-*rrnB* T1BT2 fusion fragment from pUC18Ter. This product was then ligated between the *Eco*RI and *Hin*dIII sites of pUC19, giving rise to pUC19TpTer. Last, the TpTer cassette of pUC19TpTer was excised as a *Stu*I fragment and ligated between the filled-in *Eco*RI sites of p34E-Km, thereby replacing the Km^R^ cassette.

To construct pSHAFT2, the *catA2* region of pSHAFT was amplified with a pair of primers, pSHOOTERfor2 and pSHOOTERrev. The 871 bp product was digested with *Bam*HI and *Sal*I and ligated between the *Bgl*II and *Sal*I sites of pSHAFT, thereby replacing the Ω-Cm interposon.

To construct pSHAFT3, first, a double-stranded oligonucleotide was generated by annealing oligonucleotides pSHAFT3MCSfor and pSHAFT3MCSrev. This was achieved by combining 45 μM of each oligonucleotide in 1 × KOD DNA polymerase reaction buffer (Millipore) and 1 mM MgCl_2_, incubating at 90 °C for 10 min followed by incubation at room temperature for 1 h and subsequent purification of the double-stranded oligonucleotide. pSHAFT2 was restricted with *Not*I and *Eco*RI and ligated to the double-stranded oligonucleotide, to generate pSHAFT3.

To construct pSHAFT-GFP, an 866 bp DNA fragment containing the *gfp* coding sequence and Shine-Dalgarno was amplified from pBHR1-GFP with primers GFPfor and GFPrev, purified and digested with *Bam*HI and *Sal*I. This was then ligated between *Bgl*II-*Sal*I sites of pSHAFT to replace the 3.7 kb Ω-Cm fragment of this vector.

The sequences of p34E-Km, p34E-Cm2, p34E-TpTer, pSHAFT, pSHAFT2, pSHAFT3 and pSHAFT-GFP were deposited in GenBank database, and given the accession numbers KX485327, KX485328, KX485329, KX485330, KX485332, KX485333 and KX485331, respectively. As we observed that the nucleotide sequence of the *dfrB2* cassette in p34E-Tp as deposited in the database is incorrect, we have also submitted an amended sequence of this plasmid that has been assigned accession number KX485326. pUC18Ter and pUC19TpTer were assigned accession numbers KX527623 and KX527624, respectively.

### 2.4 Gene replacement in *B. cenocepacia*

To inactivate chromosomal genes in *Burkholderia* using pSHAFT2 or pSHAFT3, the antibiotic counter-selection-based strategy used for allelic replacement by pSHAFT was employed (Agnoli et al., 2006). For isolation of a *B. cenocepacia* BCAM0195::Tp mutant, a 3.64 kb DNA fragment containing the BCAM0195 gene orthologue in strain H111 was amplified from a boiled lysate using primers BCAM0195for and BCAM0195rev, restricted with *Hin*dIII and *Bam*HI and ligated between the corresponding sites of pBBR1MCS-1, generating pBBR1-BCAM0195’. The cloned 3.11 kb gene fragment was then disrupted by ligation of the p34E-Tp-derived Tp^R^ cassette as a *Sma*I-fragment between two *Pml*I sites located 1.58 kb apart within BCAM0195, generating pBBR1-ΔBCAM0195'::Tp. The 2.04 kb ΔBCAM0195'::Tp allele was transferred to pSHAFT2 as a *Xho*I-*Xba*I fragment to give pSHAFT2-ΔBCAM0195'::Tp. This plasmid was conjugated into *B. cenocepacia* H111 using *E. coli* donor strain S17-1(λpir) according to Herrero et al. (1990) and de Lorenzo and Timmis (1994), and *B. cenocepacia* were selected on M9-glucose agar containing trimethoprim. Exconjugants were patched onto the same medium and also LB agar containing chloramphenicol. Exconjugants that were chloramphenicol-sensitive were identified as candidate ΔBCAM0195::Tp mutants. Due to the loss of short regions of DNA at the 5′ and 3′ ends of BCAM0195 during the construction of pSHAFT2-ΔBCAM0195'::Tp, potential ΔBCAM0195::Tp mutants could be verified by PCR using the original BCAM0195for and BCAM0195rev primers, as they annealed to genomic sequences located a short distance either side of the region of DNA that was also present in pSHAFT2-ΔBCAM0195'::Tp.

For isolation of a BCAL1709::TpTer mutant in *B. cenocepacia* AHA27, a 1.354 kb DNA fragment containing the 3′ region of the BCAL1709 orthologue was amplified from *B. cenocepacia* Pc715j genomic DNA using primers BCAL1709for and BCAL1709rev, restricted with *Xba*I *and Xho*I, and the resulting 1.297 kb amplicon was ligated between the corresponding sites of pSHAFT-GFP to give rise to pSHAFT-GFP-BCAL1709. The BCAL1709 gene was then disrupted by ligation of the p34E-TpTer-derived Tp^R^ cassette as a *Sma*I fragment into a unique *Zra*I site within BCAL1709, resulting in pSHAFT-GFP-BCAL1709::TpTer. This plasmid was then conjugated into AHA27 and exconjugants containing the BCAL1709::TpTer allele within the genome were selected for as described for construction of the BCAM0195::Tp mutant. The presence of *gfp* on pSHAFT-GFP causes recipient colonies to fluoresce under UV light, and was used to distinguish between recombinants that arose through a single crossover (i.e. plasmid integration - fluorescent) and a double crossover (i.e. allelic replacement - non-fluorescent). 50 trimethoprim-resistant exconjugants were patched on duplicate IST agar plates containing trimethoprim. One of each pair of plates was exposed to UV light on a transilluminator in the dark to identify non-fluorescent candidate AHA27 BCA1709::TpTer mutants. Candidate mutants from the non-irradiated duplicate plate(s) were verified by PCR using primers BCAL1709forOut and BCAL1709revOut, which annealed to genomic sequences a short distance either side of the region of DNA that was also present in pSHAFT-GFP-BCAL1709::TpTer.

## 3 Results and discussion

### 3.1 Construction of antibiotic resistance cassettes for genetic manipulation of *Burkholderia*

To facilitate the generation of mutants in *Burkholderia* through insertional inactivation of chromosomal target genes by antibiotic resistance markers and the construction of useful plasmid vectors that would allow an investigation into gene function and control in members of this genus, we made derivatives of the cassette cloning vector, p34E (Tsang et al., 1991), harbouring the kanamycin- (*aphA2*), chloramphenicol- (*catA2*) and trimethoprim- (*dfrB2*), resistance genes.

**Fig. 1:**
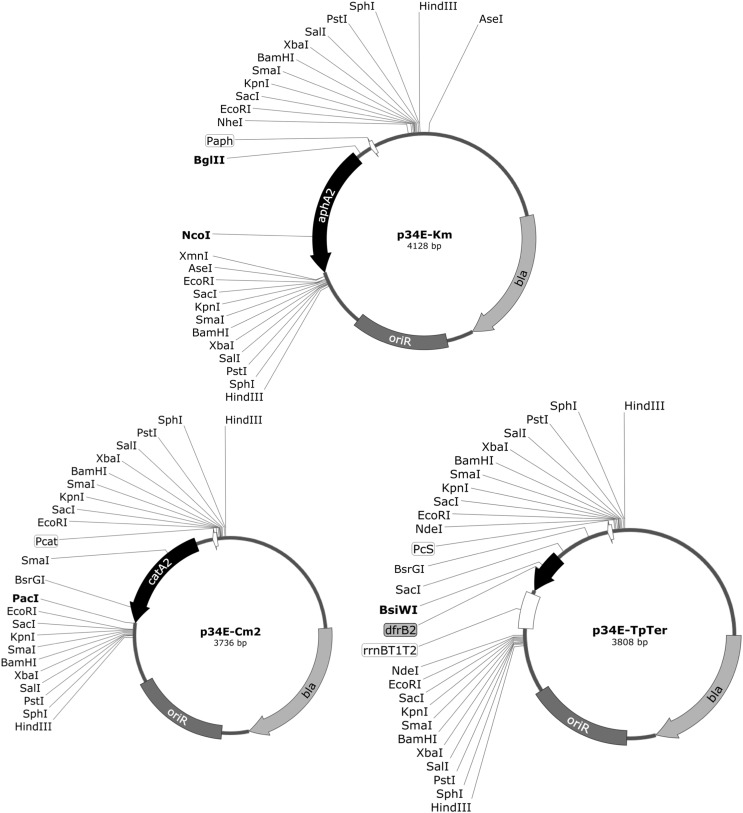
Maps of novel antibiotic-cassette vectors. The location of antibiotic resistance-conferring genes, *bla* (ampicillin-resistance), *aphA2* (kanamycin-resistance), *catA2* (chloramphenicol-resistance) and *dfrB2* (trimethoprim-resistance), and the plasmid ColE1 origin of replication (oriR) are indicated for plasmids p34E-Km (top), p34E-Cm2 (left) and p34E-TpTer. Dual-cutting restriction sites that can be utilised to excise the antibiotic resistance cassette are also shown, as are internal sites that can be used for determination of the orientation of the cassette following insertion into a target gene. Restriction sites that occur only once in each plasmid are shown in bold. Note that additional sites for *Ase*I, *Bsr*GI, *Nhe*I and *Xmn*I occur in the backbone of all three vectors that for clarity are not shown. Promoters for the cassette antibiotic-resistance genes are shown (P_aph_, P_cat_, P_cS_). Maps created with SnapGene® software (from GSL Biotech; available at snapgene.com).

First, p34E-Km was constructed by replacing the trimethoprim-resistance (Tp^R^) cassette of p34E-Tp with the Tn*5*-derived *aphA2* (*aph(3′)-II*) gene from pKNOCK-Km (Fig. 1). Although a similar kanamycin-resistance cassette (Km^R^) plasmid, p34S-Km (which contains the Tn*903*-derived *aph(3′)*-*Ia* gene), has been previously described (Dennis and Zylstra, 1998a), the Tn*5*-derived antibiotic resistance marker in p34E-Km offers the advantage that it makes available the *Hin*dIII and *Sma*I sites flanking the cassette (as they do not cut within the *aphA2* gene) and also contains flanking *Eco*RI sites. Both types of cassette are of a similar size (~ 1.3 kb). A shorter variant of the Tn903-based Km^R^ cassette lacking the internal *Hin*dIII and *Sma*I sites was subsequently constructed, but it lacks the flanking *Eco*RI sites and the possibility for directional cloning associated with p34E-Km (Dennis and Zylstra, 1998b).

Most plasmids used for genetic manipulation that specify chloramphenicol resistance (Cm^R^) harbour the Tn*9*-derived *catA1* gene (or one that is closely related) that specifies an enzyme belonging to group 1 of the type A chloramphenicol acetyltransferases (CATs) (Shaw, 1983, Schwarz et al., 2004). A notable exception is mini-Tn*5*Cm, which contains the *catA2* gene (de Lorenzo et al., 1990). Pertinently, chloramphenicol resistance conferred by this mini-transposon is selectable in single copy in *B. cenocepacia* (Farmer and Thomas, 2004). However, the *catA2* gene specifying this resistance is located on a large DNA fragment (the ~ 3.7 kb Ω-Cm interposon) that was used to assemble the transposon and is not available as a small cassette (Fellay et al., 1987, de Lorenzo et al., 1990). The Ω-Cm interposon includes a 2.8 kb DNA fragment derived from the cloning vector pKT210 which contains the 214 codon *catA2* ORF and several other predicted ORFs found on the naturally occurring BHR plasmid pSa (Bagdasarian et al., 1981, Tait et al., 1982, Shaw, 1983, Fellay et al., 1987). In order to generate a compact Cm^R^ cassette containing an efficiently expressed *cat* gene, the *catA2* gene contained on pSHAFT (a suicide plasmid derived from pUTmini-Tn*5*Cm (see below)), was modified to incorporate a synthetic σ^70^-dependent promoter upstream of the *catA2* TIR. The modified *catA2* gene was used to replace the Km^R^ cassette of p34E-Km, to generate p34E-Cm2 (Fig. 1). The Cm^R^ cassette in p34E-Cm2, can be transferred to other vectors using any of the flanking restriction sites except *Sma*I (Fig. 1), although even here, selection for chloramphenicol resistance would permit selection for the products of a tripartite ligation between the target vector and the two *Sma*I-generated cassette fragments. As an alternative, either of the blunt end-generating *Sac*I isoschizomers, *Eco*53kI or *Ecl*136II, may be used.

The most similar vectors available as sources of a Cm^R^ cassette are p34S-Cm and -Cm2 (Dennis and Zylstra, 1998a, Dennis and Zylstra, 1998b). Rather than the *catA2* gene present in p34E-Cm2, these other plasmids contain the Tn*9*-derived *catA1* gene and the cassettes are slightly longer (918 bp rather than 823 bp). p34S-Cm contains an internal *Eco*RI site whereas in p34S-Cm2 this site has been removed. However, unlike p34E-Cm2, p34S-Cm2 lacks flanking *Eco*RI sites.

The trimethoprim-resistance gene (*dfrB2*) present on the cassette vectors p34E-Tp and p34S-Tp is under control of the very strong PcS integron promoter (also known as *P*1) (Lévesque et al., 1994, DeShazer and Woods, 1996, Dennis and Zylstra, 1998a, Jové et al., 2010). We have observed that insertion of the Tp^R^ cassette in certain orientations in some plasmid vectors is not possible (see below) and when used in allelic replacement experiments it can result in impaired growth of the resultant mutants, presumably due to overexpression of chromosomal genes located downstream of the integrated cassette (S.S. and M.S.T., unpublished observations). To circumvent this problem, we modified the Tp^R^ cassette by placing the efficient T1 T2 transcription terminators from the *E. coli rrnB* ribosomal RNA operon, downstream of the *dfrB2* coding sequence (Brosius, 1984, Orosz et al., 1991). To do this, we started with pEA302T, a plasmid into which a 500 bp *Eco*RI fragment containing the 5S rRNA gene and the T1 T2 terminators was cloned in order to prevent read through transcription of the replication region (Amann et al., 1983). A DNA fragment containing only the *rrnB* T1 T2 terminators was amplified using primers that also incorporated additional restriction sites at the flanking regions of the fragment, and was subsequently transferred to pUC18 to give pUC18Ter.

The *rrnB* terminators (from pUC18Ter) were then fused to the *dfrB2* gene (from p34E-Tp) by the megaprimer variation of the SOE PCR technique (Perrin and Gilliland, 1990), and the amplicon was ligated into pUC19, giving rise to pUC19TpTer. Although the TpTer DNA fragment in pUC19TpTer can be transferred as a *Stu*I or *Nde*I cassette, to increase its versatility, it was transferred into p34E-Km, substituting for the Km^R^ cassette. The resultant plasmid, p34E-TpTer, is analogous to p34E-Tp but has strong transcription termination signals located downstream of the *dfrB2* gene and includes the addition of *Nde*I sites in the flanking MCSs (Fig. 1).

### 3.2 Construction of allelic replacement vectors, pSHAFT2 and pSHAFT3, for generation of marked mutants in *Burkholderia*

**Fig. 2:**
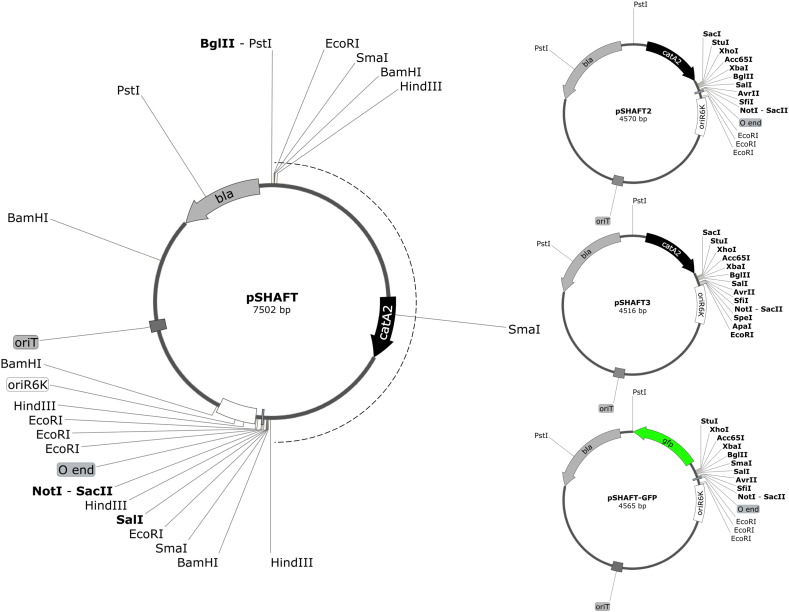
Maps of the gene replacement vectors of the pSHAFT-series utilised for marked mutagenesis in *Burkholderia*. The location of antibiotic-resistance conferring genes, *bla* (ampicillin-resistance) and *catA2* (chloramphenicol-resistance), RP4 origin of transfer (*oriT*), R6K origin of replication (*oriR6K*) and GFP-encoding gene (*gfp*) are indicated for pSHAFT, (left), pSHAFT2 (top right), pSHAFT3 (centre right) and pSHAFT-GFP (bottom right). The transcriptional orientation for each gene and restriction sites within the multiple cloning site of each vector are shown. Additional restriction sites in pSHAFT that flank the Ω-Cm interposon (dashed line) are also indicated. Restriction sites that occur only once in each plasmid are shown in bold. Maps created with SnapGene® software (from GSL Biotech; available at snapgene.com).

We sought to improve upon existing vectors used for allelic replacement in the Bcc. A useful vector for insertional inactivation of chromosomal genes with selective markers (usually antibiotic resistance cassettes) is pSHAFT, an R6K-based suicide plasmid (Fig. 2) (Agnoli et al., 2006). This vector was derived from the transposon delivery plasmid, pUTmini-Tn*5*Cm (de Lorenzo et al., 1990), by deletion of the *tnp* (transposase) gene and one of the 19 bp repeat sequences (the ‘I end’) that flank mini-Tn*5*Cm, and therefore it cannot be mobilised in the presence of transposase provided in *trans*. pSHAFT is also devoid of the *pir* gene, which specifies the plasmid replication initiator protein, π, and can therefore only replicate in bacteria containing the *pir* gene, such as *E. coli* CC118(λpir) (Herrero et al., 1990, Rakowski and Filutowicz, 2013). Along with the chloramphenicol-resistance marker carried by mini-Tn*5*Cm, pSHAFT also contains the origin of transfer (*oriT*) from RP4 (RK2) that allows for efficient conjugal transfer of the plasmid to a variety of Gram-negative bacterial species. Following transfer of a Bcc gene (or gene fragment) to pSHAFT, the gene is disrupted by insertion an antibiotic-resistance cassette (other than Cm^R^) that is selectable in the Bcc. The plasmid is then introduced into a Bcc strain and selection for the presence of the antibiotic-resistance cassette (Ab^R^) is applied. As the plasmid cannot replicate in Bcc, only recombinants are obtained in which the plasmid has recombined with the host genome at the locus that is homologous to the Bcc DNA present on the plasmid. This may result in integration of the plasmid into the genome (single crossover, Cm^R^ Ab^R^) or allelic replacement (double crossover, Cm^S^ Ab^R^).

However, the major drawback with pSHAFT is the limited number of unique restriction sites that can be used for inserting mutant alleles for subsequent chromosomal targeting. One reason for this is the duplication of restriction sites either side of the Cm^R^ element during the assembly of mini-Tn*5*Cm in the progenitor plasmid pUT (de Lorenzo and Timmis, 1994). Moreover, as discussed above, the cassette specifying resistance to chloramphenicol is unnecessarily large, as mini-Tn*5*Cm was originally constructed by inserting the ~ 3.7 kb Ω-Cm interposon between the 19 bp I and O ends of Tn*5* carried by pUT (de Lorenzo et al., 1990).

To improve the utility of pSHAFT, the vector was modified by replacing the interposon with a much shorter DNA fragment containing the *catA2* gene under control of a synthetic σ^70^-dependent promoter as also incorporated in p34E-Cm2, thereby decreasing the plasmid size from 7.5 to 4.6 kb. The new plasmid, pSHAFT2, contains 11 unique restriction sites located downstream of the *catA2* gene, one of which (*Stu*I) allows the cloning of blunt-ended fragments, while the *Bgl*II site can also accommodate *Bam*HI-generated fragments. At the *catA2*-distal end of the MCS are three closely spaced *Eco*RI sites that can be considered as an additional single unique site for cloning purposes (Fig. 2).

The versatility of pSHAFT2 was then improved by replacing the region extending from the *Not*I site in the MCS to the most distant of the three *Eco*RI sites by a double-stranded oligonucleotide that contained internal *Spe*I and *Apa*I sites. This also resulted in a net loss of two of the three *Eco*RI sites present in pSHAFT2 and removal of the mini-Tn*5* O end that originated from the progenitor plasmid of pSHAFT. The new plasmid, pSHAFT3 (4.5 kb), is shown in Fig. 2.

### 3.3 Construction of an allelic replacement vector, pSHAFT-GFP, that allows fluorogenic detection of integration events in *Burkholderia species* during generation of marked mutants

To allow for inactivation of chromosomal genes where the *Burkholderia* strain already harbours a chloramphenicol resistance marker or to disrupt chromosomal genes with a Cm^R^ cassette we constructed pSHAFT-GFP (Fig. 2). This plasmid is analogous to pSHAFT2 but with the *catA2* gene replaced by the *gfp* gene, which therefore allows for fluorogenic detection of recombinants containing the genomically integrated vector. The forward primer used to amplify the *gfp* gene specified recognition sites for the restriction enzymes *Sal*I, *Sma*I, *Bgl*II, *Xba*I, *Kpn*I, *Xho*I and *Stu*I and an artificial promoter containing the consensus − 35 and − 10 elements for σ^70^-dependent promoters, whereas the reverse primer specified only a *Bam*HI site. This vector is used in the same way as pSHAFT2 except for the fact that single and double crossover recombinants harbouring the selectable marker used to inactivate the target chromosomal gene are distinguished from each other by screening colonies for the absence of yellow-green fluorescence. Although this usually requires exposure to UV in the dark (a UV transilluminator works well in this regard), in some mutagenesis experiments the presence of the GFP marker can be discerned without recourse to UV exposure by the yellow-green colour of the colonies.

### 3.4 Isolation of marked mutants in *B. cenocepacia* and related species using the pSHAFT-vector series

**Fig. 3:**
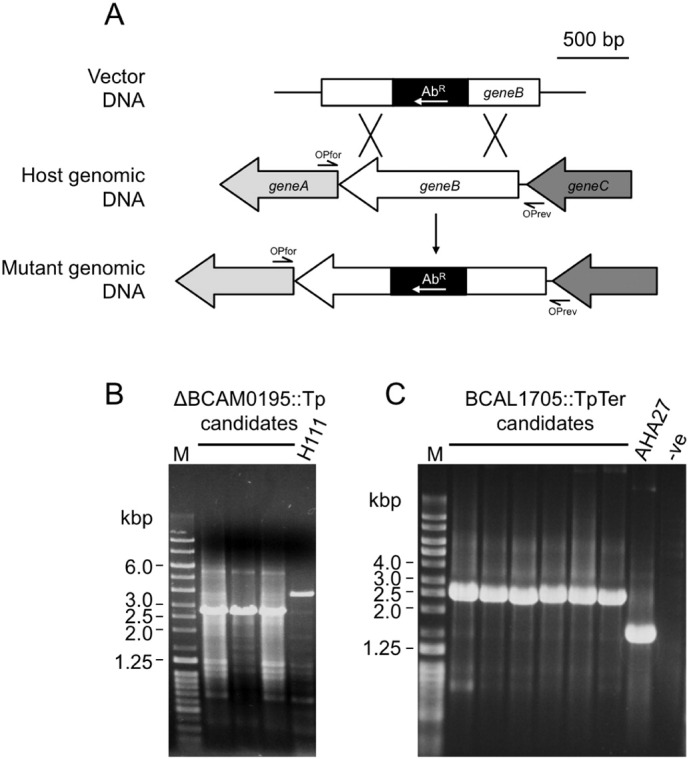
Generation of marked mutants in *B. cenocepacia* using pSHAFT2 and pSHAFT-GFP derivatives. (A) ≥ 1.0 kb of DNA containing the target gene (or gene fragment) is cloned into a pSHAFT vector and then disrupted by insertion of an antibiotic resistance cassette, ensuring there is at least 0.5 kb of homology between the cloned DNA target region and the chromosome on either side of the cassette. Following transfer of the pSHAFT-derived construct into *Burkholderia*, double crossover recombinants are selected for based on their resistance to the antibiotic specified by the antibiotic resistance cassette, and either sensitivity to chloramphenicol (pSHAFT2 and pSHAFT3) or the absence of fluorescence (pSHAFT-GFP). Candidate mutants are then verified by PCR using primers that anneal to genomic sequences located either side of the region cloned into the allelic replacement vector (‘outside’ primers), indicated as OPfor and OPrev. Drawn to scale. (B) PCR screening of candidate H111-ΔBCAM0195::Tp mutants following allelic replacement with pSHAFT2-ΔBCAM0195’::Tp. (C) PCR screening of candidate AHA27-BCAL1709::TpTer mutants following allelic replacement with pSHAFT-GFP-BCAL1709::TpTer.

To inactivate chromosomal genes in *Burkholderia* using the pSHAFT-vector series, DNA fragments of ≥ 1.0 kb containing the target gene (or gene fragment) are inserted into the MCS and then subsequently disrupted by insertion of an antibiotic resistance cassette (usually Tp^R^, Km^R^ or Tc^R^) such that at least 0.5 kb of homology occurs between the cloned DNA target region and the chromosome either side of the lesion (Fig. 3A). Depending on the availability of restriction sites, in some cases we have found it more convenient to first clone the target DNA sequence into a general-purpose plasmid vector and then introduce the antibiotic resistance cassette before transferring the disrupted DNA fragment to the suicide vector. In using these plasmids to inactivate chromosomal genes in *Burkholderia*, we have observed that the Tp^R^ cassette can often only be inserted into target genes in one orientation, such that transcription is directed towards the *catA2* gene, and away from the origin of replication. We assume this is due to transcriptional destabilisation of plasmid replication functions (Gentz et al., 1981, Stueber and Bujard, 1982, Stassi and Lacks, 1982) as this problem does not occur with the TpTer cassette (our unpublished observations).

Once the disrupted gene or gene fragment has been introduced into pSHAFT2, it is transferred to *Burkholderia* and selection is applied for the antibiotic resistance marker that was used to disrupt the gene or gene fragment present in the suicide vector, thus identifying strains that have integrated the disrupted allele into the genome by homologous recombination. Recombinants are of two types: those that are the result of a single crossover, in which the entire plasmid has integrated into the genome at the target gene locus, and those that are the result of a double crossover in which only the mutant allele has been transferred to the genome in place of the original wild type gene (Fig. 3A). In the former, wild type and mutated copies of the gene (or gene fragment) are present in the genome of the recipient bacterium, which will also specify increased resistance to chloramphenicol due to the integrated vector. These are identified by ‘patching’ out recombinants on LB agar containing 50 μg/ml chloramphenicol. Chloramphenicol-sensitive recombinants are screened for the presence of the mutated allele (and absence of the wild type allele) by PCR using primers that anneal to genomic sequences flanking the region that was originally cloned in the suicide vector.

We have successfully used pSHAFT2 to generate several marked mutants within Bcc species. This included the insertional inactivation of the I35_0520 gene in *B. cenocepacia* strain H111 (the orthologue of BCAM0195 in strain J2315 and will be referred to as such hereon), which was disrupted by the Tp^R^ cassette derived from p34E-Tp. BCAM0195 is predicted to encode a non-ribosomal peptide synthetase (NRPS) of unknown function. During the isolation of the ΔBCAM0195::Tp mutant, we observed that out of 50 trimethoprim resistant exconjugants, 16 were chloramphenicol sensitive. Three of these were verified as ΔBCAM0195::Tp mutants by PCR, where the DNA fragment amplified from BCAM0195 in the mutants was ~ 900 bp smaller than the wild-type, due to replacement of a large segment of BCAM0195 by the Tp^R^ cassette in the ΔBCAM0195::Tp allele (Fig. 3B). We have also used this plasmid to introduce an *orbI*::Tp allele into the genome of *B. lata* using the same selection conditions employed for allelic replacement in *B. cenocepacia* (results not shown). The applicability of the pSHAFT vectors to other non-Bcc species within the genus was established by generating a type VI secretion system mutant of *B. thailandensis* (a member of the ‘pseudomallei’ group) in an analogous fashion using pSHAFT3 as the vector and the TpTer cassette to disrupt the *tssK* gene (results not shown).

To demonstrate the utility of pSHAFT-GFP it was used to inactivate the orthologue of the *B. cenocepacia* J2315 BCAL1709 gene that is present in the siderophore-negative *B. cenocepacia* mutant AHA27. AHA27 is a derivative of *B. cenocepacia* strain Pc715j that contains a mini-Tn*5*CmlacZYA insertion in the *pobA* gene encoding a phosphopantetheinyl transferase (PPTase) required for activating NRPS enzymes, and thereby specifies higher levels of resistance to chloramphenicol than the parent strain (Asghar et al., 2011). Based on amino acid sequence alignment, BCAL1709 is very likely to encode a putative TonB-dependent receptor thought to be involved in the uptake of an unknown xenosiderophore complexed with ferric iron (M.S.T., unpublished results). Following cloning of a BCAL1709 gene fragment in pSHAFT-GFP and its disruption with the Tp^R^ cassette derived from p34E-TpTer, the resultant plasmid (pSHAFT-GFP-BCAL1709::TpTer) was introduced into AHA27 and candidate BCAL1709 mutants were selected by screening trimethoprim-resistant exconjugants for the absence of GFP-mediated fluorescence. Among 50 ex-conjugants that were screened in this way, 6 non-fluorescent recombinants were identified which were subsequently verified as BCAL1709 mutants by PCR, as indicated by a ~ 900 bp increase in the size of the DNA fragment amplified from the BCAL1709 locus due to the presence of the Tp^R^ cassette (Fig. 3C).

It should also be noted that use of the Tp^R^ cassette in allelic replacement experiments is also applicable to *Burkholderia* strains that exhibit higher levels of intrinsic resistance to trimethoprim, such as members of the *B. cenocepacia* ET-12 lineage, i.e. strains J2315 and K56-2. Due to the highly active promoter located upstream of the *dfrB2* ORF, expression of the gene is sufficiently strong to allow its selection in such strains by increasing the concentration of trimethoprim in the medium, and we have used pSHAFT2 to transfer Tp^R^ cassette-disrupted iron acquisition and type VI secretion system genes into K56-2 (unpublished results).

## 4 Conclusion

To conclude, we have constructed a series of versatile suicide vectors and antibiotic resistance cassettes that allow for the efficient and simple generation of marked mutants in *Burkholderia* species. We have improved upon our original vector pSHAFT by reducing the size of the Cm^R^ marker and increasing the availability of unique restriction site for cloning in vectors pSHAFT2 and pSHAFT3. The versatility of this plasmid series has been further enhanced by incorporating a fluorescent marker in pSHAFT-GFP to provide an alternative means of distinguishing recombinants that have undergone allelic replacement events from those in which the suicide vector remains integrated in the genome. All vectors described here are a useful addition to the molecular toolkit required for the manipulation and subsequent characterization of important genes in not only *Burkholderia* species, but potentially other Proteobacteria, such as enterobacteria and pseudomonads, where they may be used in combination with the antibiotic-resistance cassettes described herein or other available antibiotic-resistance markers.

## Appendix A Supplementary data

Bacterial strains, plasmids and oligonucleotides. Image 1
