# Supplementary material for: An efficient system for the generation of marked genetic mutants in members of the genus Burkholderia
Source: Plasmid. 2017 Jan;89:49–56. doi: 10.1016/j.plasmid.2016.11.002 (PMC5312678; doi:10.1016/j.plasmid.2016.11.002)
Supplement: Supplementary file 1 — Bacterial strains, plasmids and oligonucleotides. [file mmc1.docx]

**Supplemental**

| **Table S1.** Bacterial strains and plasmids used in this study | | |
| --- | --- | --- |
| **Strain or plasmid** | **Genotype or description** | **Source or reference** |
| **Bacterial strains:** | | |
| ***E. coli*** |  |  |
| JM83 | F^−^ *ara* Δ(l*ac-proAB*) *rpsL ϕ*80d*lacZ*ΔM15 (Sm^R^) | Yanisch-Perron et al., 1985 |
| SM10(λpir) | *thi-1 thr leu tonA lacY supE* *recA*::RP4-2-Tc::Mu (Km^R^)(λpir) | Simon et al., 1983 |
| S17-1(λpir) | *thi proA hsdR recA* RP4-2-*tet*::Mu-1 *kan*::Tn7 integrant (Tp^R^, Sm^R^) (λpir) | Simon et al., 1983 |
|  |  |  |
| ***B. cenocepacia*** | |  |
| Pc715j | CF isolate, prototroph | McKevitt et al., 1989 |
| H111  J2315 | CF isolate, prototroph  CF isolate | Römling et al., 1994  Holden et al., 2009 |
| K56-2  AHA27 | CF isolate, prototroph  Pc715j-*pobA*::mini-Tn*5*CmlacZYA | Darling et al., 1998; Mahenthiralingam et al., 2000  Asghar et al., 2011 |
| H111-BCAM0195::Tp | H111 with *dfrB2* cassette inserted in BCAM0195 (Tp^R^) | This study |
| AHA27-BCAL1709::TpTer | AHA27 with *dfrB2* cassette containing *rrnB* T1T2 terminators inserted in BCAL1709 (Tp^R^) | This study |
| ***B. lata*** |  |  |
| 383  ***B. thailandensis*** | Soil isolate, prototroph (also known as (ATCC 17760, NCIB 9087, LMG 22485) | Stanier et al., 1966; Vanlaere et al., 2009 |
| E264 | Rice paddy isolate, prototroph | Brett et al.,1998 |
| **Plasmids:** |  |  |
| pEA302T | *E. coli*-specific vector containing phage λ *c*I under P*_tac_* with *rrnB* T1T2 terminators (Ap^R^, Tc^R^) | Amann et al., 1983 |
| pBBR1MCS-1 | Mobilizable BHR cloning vector, pBBR1-replicon (Cm^R^) | Kovach et al., 1994 |
| pUC18 | *E. coli*-specific cloning vector (Ap^R^) | Yanisch-Perron et al., 1985 |
| pUC19 | *E. coli*-specific cloning vector (Ap^R^) | Yanisch-Perron et al., 1985 |
| pUC18Ter | pUC18 containing *rrnB* T1T2 terminators (Ap^R^) | This study |
| pUC19TpTer | pUC19 containing *dfrB2* gene fused to *rrnB* T1T2 terminators (Ap^R^, Tp^R^) | This study |
| pKNOCK-Km | Mobilizable suicide vector containing *aphA2* gene (Km^R^) | Alexeyev, 1999 |
| p34E-Tp | p34E containing *dfrB2* gene (Ap^R^, Tp^R^) | DeShazer and Woods, 1996 |
| p34E-Km | p34E containing *aphA2* gene from pKNOCK-Km (Ap^R^, Km^R^) | This study |
| p34E-Cm2 | p34E containing *catA2* gene from pSa with synthetic promoter (Ap^R^, Cm^R^) | This study |
| p34E-TpTer | p34E containing *dfrB2* gene fused to *rrnB* T1T2 terminators (Ap^R^, Tp^R^) | This study |
| pBHR1-GFP | pBHR1 containing *gfp* gene from pQBI-T7-GFP (Cm^R^, Km^R^) | Stevens et al., 2005 |
| pSHAFT | Suicide vector derived from pUTmini-Tn*5*Cm containing deletion of *Bgl*II fragment harboring *tnp* gene and I end of mini-Tn*5*Cm, R6K-derived replicon, *ori*T^+^(Ap^R^, Cm^R^) | Shalom, 2002; Agnoli et al., 2006 |
| pSHAFT-GFP | pSHAFT with 3.7 kb *Ω*-Cm interposon replaced by *gfp* gene from pBHR1-GFP | This study |
| pSHAFT2 | pSHAFT with 3.7 kb *Ω*-Cm region replaced by *catA2* gene driven by synthetic promoter and additional unique restriction sites at 3’ end (Ap^R^, Cm^R^) | This study |
| pSHAFT3 | pSHAFT2 derivative with deletion of two *Eco*RI sites, addition of *Apa*I and *Spe*I sites and removal of the mini-Tn*5* O end (Ap^R^, Cm^R^) | This study |
| pBBR1-BCAM0195’ | pBBR1MCS-1 containing *B. cenocepacia* H111 BCAM0195 gene fragment, lacking 0.55 kbp at the 3’ end (Cm^R^) | This study |
| pBBR1-BCAM0195’::Tp | pBBR1-BCAM0195’ with *dfrB2* gene replacing a 1.58 kb segment of BCAM0195’ (Cm^R^, Tp^R^) | This study |
| pSHAFT2-BCAM0195’::Tp | pSHAFT2 containing the *Xho*I-*Xba*I BCAM0195’::Tp fragment from pBBR1-BCAM0195’::Tp (Ap^R^, Cm^R^, Tp^R^) | This study |
| pSHAFT-GFP*-*BCAL1709 | pSHAFT-GFP containing *B. cenocepacia* Pc715j BCAL1709 gene (Ap^R^, Cm^R^) | This study |
| pSHAFT-GFP*-*BCAL1709::TpTer | pSHAFT-GFP*-*BCAL1709 with *dfrB2-rrnB* T1T2 cassette inserted in BCAL1709 (Ap^R^, Cm^R^, Tp^R^) | This study |
|  | | |
| **Abbreviations:** Ap^R^, ampicillin-resistant; Cm^R^, chloramphenicol-resistant; Km^R^, kanamycin-resistant; Sm^R^, streptomycin-resistant; Tc^R^, tetracycline-resistant; Tp^R^, trimethoprim-resistant; BHR, broad host range. | | |

| **Table S2.** Primers used in this study | |
| --- | --- |
| **Primer ID** | **Primer sequence^a,b^** |
|  | |
| p34E-Cmfor2 | 5’-GCGCGAATTCTTGACAATTAAGCCCGTATATGGTATTATTA  CTGAAT |
| p34E-Cmrev | 5’-GCGCGAATTCCCGGATACGGTGGCTTAAAT |
| rrnBterfor | 5'-CGCGGATCCAATTGAGAGTAGGGAACTGCCAGGCA |
| rrnBterrev | 5'-GCGCAAGCTTCTCGAGGGTACCGAGCTCGAATTCTTGTAG  ATATGACGACAGGA |
| Tp(forward) | 5'-GCGGAATTCAGGCCTCATATGCACGAACCCAGTTGACAT |
| Tp(reverse) | 5'-CTGGCAGTTCCCTACTCTCTTAGGCCACACGTTCAAGTGC |
| rrn(R) | 5'-GACAAGCTTAGGCCTCATATGGTAGATATGACGACAGGAA  GAG |
| pSHOOTERfor2 | 5’-GCGCGGATCCTTGACAATTAAGCCCGTATATGGTATTATT  ACTGAAT |
| pSHOOTERrev | 5’-GCGCGTCGACAAAGATCTAATCTAGAAAGGTACCAACTCG  AGAAAGGCCTAAGAGCTCCCGGATACGGTGGCTTAAAT |
| pSHAFT3MCSfor | 5’-GGCCGCAAACTAGTAAGGGCCCAAG |
| pSHAFT3MCSrev | 5’-AATTCTTGGGCCCTTACTAGTTTGC |
| GFPfor | 5’-GCGCGTCGACAACCCGGGAAAGATCTAATCTAGAAAGGTA  CCAACTCGAGAAAGGCCTTTGACATTTGCAGATTCGCCTTC  TATAATAATTCGCCCTTCCCCTGTAGAAATAATTTTG |
| GFPrev | 5’-CTTTGTTAGCAGCCGGATCC |
| BCAM0195for | 5’-GCGCAAGCTTATGAGCGGCCTGCTCGATCA |
| BCAM0195rev | 5’-GCGCGGATCCACGTCTTCACCACGCGGGTT |
| BCAL1709for | 5’-GCGCTCTAGAGCTGCTGCAGTTCGAATACG |
| BCAL1709rev | 5’-CGTCGCATTCGCGTAGTAGT |
| BCAL1709forOut | 5’-CGGAAAACTTCGGACATGTG |
| BCAL1709revOut | 5’-GCGAGGTCAGAACTTGTATG |

^a^Sequences specifying restriction endonuclease cleavage sites are underlined.

^b^Sequences corresponding to the -35 and -10 elements of the artificial promoters introduced upstream of the *catA2* and *gfp* genes are enclosed in boxes. The former is an ‘extended’ -10 promoter (Bown et al., 1997).

**References**

Alexeyev, M.F., 1999. The pKNOCK series of broad-host-range mobilizable suicide vectors for gene knockout and targeted DNA insertion into the chromosome of gram-negative bacteria. Biotechniques 26 (5), pp. 824-828.

Bown, J., Barne, K., Minchin, S., Busby, S., 1997. Extended -10 promoters. In: Eckstein,

F., Lilley, D.M.J. (Eds.), Nucleic Acids Mol. Biol. Springer, Berlin, pp. 41-52.

Brett, P.J., DeShazer, D., Woods, D.E., 1998. *Burkholderia thailandensis* sp. nov., a *Burkholderia pseudomallei*-like species. Int. J. Syst. Bacteriol. 48 (1), pp. 317-320.

Darling, P., Chan, M., Cox, A., Sokol, P.A. 1998. Siderophore production by cystic fibrosis isolates of *Burkholderia cepacia*. Infect. Immun*.*, 66 (2), pp. 874-877.

Holden, M.T.G. et al., 2009. The genome of *Burkholderia cenocepacia* J2315, an epidemic pathogen of cystic fibrosis patients. J. Bacteriol*.* 191 (1), pp.261-277.

Kovach, M.E., Phillips, R.W., Elzer, P.H., Roop, R.M., Peterson, K.M., 1994. pBBR1MCS: a broad-host-range cloning vector. Biotechniques 16 (5), pp. 800-802.

Mahenthiralingam, E. et al., 2000. Diagnostically and experimentally useful panel of strains from the *Burkholderia cepacia* complex. J. Clin. Microbiol. 38 (2), pp. 910-913.

McKevitt, A.I., Bajaksouzian, S., Klinger, J.D., Woods, D.E., 1989. Purification and characterization of an extracellular protease from *Pseudomonas cepacia*. Infect. Immun. 57 (3), pp. 771-778.

Römling, U. et al., 1994. Epidemiology of chronic *Pseudomonas aeruginosa* infections in cystic fibrosis. J. Infect. Dis. 170 (6), pp. 1616-1621.

Shalom, G., 2002. Ph.D. thesis. University of Sheffield, Sheffield, England.

Simon, R., Priefer, U., Pühler, A., 1983. A Broad Host Range Mobilization System for In Vivo Genetic Engineering: Transposon Mutagenesis in Gram Negative Bacteria. Bio/Technology 1 (9), pp. 784-791.

Stanier, R.Y., Palleroni, N.J., Doudoroff, M., 1966. The aerobic pseudomonads: a taxonomic study. J. Gen. Microbiol. 43 (1), pp. 159-271.

Stevens, J.M. et al., 2005. Actin-binding proteins from *Burkholderia mallei* and *Burkholderia thailandensis* can functionally compensate for the actin-based motility defect of a *Burkholderia pseudomallei bimA* mutant. J. Bacteriol*.* 187 (22), pp. 7857-7862.

Vanlaere, E. et al., 2009. Taxon K, a complex within the *Burkholderia cepacia* complex, comprises at least two novel species, *Burkholderia contaminans* sp. nov. and *Burkholderia lata* sp. nov. Int. J. Syst. Evol. Microbiol. 59 (1), pp. 102-111.

Yanisch-Perron, C., Vieira, J., Messing, J., 1985. Improved M13 phage cloning vectors and host strains: nucleotide sequences of the M13mp18 and pUC19 vectors. Gene 33 (1), pp. 103-119.
